# An Ultrasonication-Assisted Cobalt Hydroxide Composite with Enhanced Electrocatalytic Activity toward Oxygen Evolution Reaction

**DOI:** 10.3390/ma11101912

**Published:** 2018-10-09

**Authors:** Yujun Si, Chaozhong Guo, Chenglong Xie, Zhongping Xiong

**Affiliations:** 1 College of Chemistry and Environmental Engineering, Sichuan University of Science and Engineering, Zigong 643000, China; 13438027021@163.com (C.X.); xiongzhongp@163.com (Z.X.); 2 Research Institute for New Materials Technology, Engineering Research Center of New Energy Storage Devices and Applications, Chongqing University of Arts and Sciences, Chongqing 402160, China

**Keywords:** oxygen evolution reaction, electrocatalyst, cobalt hydroxide, ultrasonication assistance

## Abstract

A catalyst toward oxygen evolution reaction (OER) was synthesized by depositing cobalt hydroxide on carbon black. Ultrasonication was applied during precipitation to improve the performance of the catalyst. The ultrasonic-assisted process resulted in the refinement of the cobalt hydroxide particles from 400 nm to 50 nm, and the thorough incorporation of these particles with carbon black substrate. The resulting product exhibited enhanced OER catalytic activity with an onset potential of 1.54 V (vs. reversible hydrogen electrode), a Tafel slope of 18.18 mV/dec, and a stable OER potential at a current density of 10 mA cm^−2^, because of the reduced resistance of the catalyst and the electron transfer resistance.

## 1. Introduction

Clean and renewable energy sources are urgently needed to be developed to address the increasing energy crisis and pollution problems. Hydrogen, as a high-energy density noncarbon energy source, has been considered as a clean energy source, and its production has been one of the top research hotspots. Electrochemical water splitting is an effective approach to convert renewable energy sources to promising hydrogen energy [1,2,3,4,5]. This process involves a hydrogen evolution reaction at the cathode, and an oxygen evolution reaction (OER, 4OH^−^ → O_2_ + 2H_2_O + 4e^−^ in alkaline solution) at the anode. The OER is the major obstacle for water splitting, because this process is kinetically sluggish, involving a complex four-electron transfer with high overpotential [6,7,8]. Considerable attention has been paid to developing efficient catalysts for OER with low overpotential and high activity.

IrO_2_ and RuO_2_ have been demonstrated as the best OER electrocatalysts [9,10]. However, their large-scale applications are blocked by their high prices and limited natural reserves. The first-row transition metal oxides have been extensively investigated because of their natural abundance, low cost, environmental-friendliness, and high catalytic activity [11,12,13,14,15,16]. Studies have suggested that the oxy-hydroxides of nickel, cobalt, and iron are promising electrocatalysts for the OER. Co_3_O_4_ [17,18,19] and Co(OH)_2_ [20,21,22], synthesized by different methods have been widely investigated because of their high stability and high catalytic activity (theoretically, Co_3_O_4_ can close the OER activity of RuO_2_ and IrO_2_). However, the development of oxy-hydroxides of cobalt with high electrocatalytic activity to OER remains a major challenge because of poor electrical conductivity and limited exposed active sites.

To obtain an OER catalyst with better conductivity, transition metal oxide catalysts are mixed with conductive materials, such as carbon black, with the aid of polymeric binders in actual application. However, the utilization of insulating binders can reduce the electrocatalytic performance [23,24]. An efficient strategy to overcome this shortage is by fabricating nanoparticles of OER catalysts and naturally immobilizing the nanoparticles onto the conductive substrates. Fabrication of composites of oxy-hydroxides of cobalt with functionalized carbon-based materials (e.g., heteroatom-doped graphene and carbon nanotubes) has received special interest. This process can improve the poor conductivity of metal oxides, and the resulting catalysts also usually exhibit bifunctional catalytic activities [25,26]. The morphologies of the transition metal oxides also play a key role in the heterogeneous electrocatalytic reaction, because more catalytic active sites can be exposed to facilitate the OER by refining the particle sizes or by increasing the porosity of the catalysts with greater specific surface area [27,28].

Ultrasonication is a nonconventional technique that has been proven to be superior on operation, product selectivity, and reduced reaction time. Ultrasonication can bring instantaneous generation, and the growth and collapse of micrometer-sized bubbles in liquid medium, which is helpful for the synthesis of nanostructured materials. Recently, the use of ultrasonication in chemistry has grown significantly [29,30,31]. In the present work, we prepared cobalt hydroxide [Co(OH)_2_] by parallel flow precipitation on carbon black with the assistance of ultrasonication. The particles of Co(OH)_2_ were refined and well incorporated with the carbon black substrate, and the resulting catalyst shows enhanced OER catalytic activity.

## 2. Experimental

### 2.1. Synthesis of the Catalyst

A mixed solvent (pH 10) from 30 mL of deionized water and 30 mL of ethanol was placed in a water bath (50 °C) with an ultrasonic generator (40 kHz). Then, 2 g of carbon black (VXC72R, Cabot) was dispersed into the solvent under ultrasonication and mechanical stirring. Afterward, 30 mL of Co(NO_3_)_2_·6H_2_O solution (1.0 mol L^−1^, 0.03 mol) and 30 mL of KOH solution (2.0 mol L^−1^, 0.06 mol) were added into the mixed solvent at 1.0 mL min^−1^ by two peristaltic pumps. Then, ultrasonication and stirring were terminated, by keeping the reaction system in the water bath for another 10 h, followed by filtering and washing with deionized water until pH 7, drying at 80 °C for 10 h. The resulting catalyst was denoted as ultra-Co(OH)_2_/C. As a control, Co(OH)_2_/C was prepared by the same procedure but without the assistance of ultrasonication during precipitation.

### 2.2. Physical Characterizations

The phase structure of the catalyst was determined using an X-ray diffractometer (XRD) (DX-2600, Fangyuan, Dandong, China). The morphology was observed using scanning electron microscopy (SEM) (Gemini SEM 300, Zeiss, Oberkochen, Germany).

### 2.3. Electrochemical Measurements

The OER catalytic activity of the catalyst was evaluated by electrochemical measurements performed in 0.1 M KOH solution on a CHI760E workstation (Chenhua, Shanghai, China) with a platinum sheet as a counter-electrode, a saturated calomel electrode (SCE) as a reference electrode, and a glassy carbon electrode (Φ = 0.5 cm) coated with the catalyst as a working electrode by pipetting 10 μL of dispersion of catalyst (including commercial RuO_2_, 10 mg mL^−1^ of concentration) onto the electrode surface and naturally drying in air. The catalyst loading on the working electrode was 0.51 mg cm^−2^. Electrochemical linear sweep voltammetry (LSV) was conducted at a scan rate of 5 mV s^−1^. The OER potential was recorded by chronopotentiometry at a current density of 10 mA cm^−2^. The dispersion of catalyst was also dropped on graphite paper and carried out the chronopotentiometry test for 5 h, and then its micromorphology was observed. The electrochemical impedance spectrum (EIS) was tested over a frequency range from 10^5^ Hz to 0.1 Hz. All of the potentials were converted to reversible hydrogen electrode (RHE) using the equation *E*_RHE_ = *E*_SCE_ + 0.2415 V + 0.0591 pH (pH 13).

## 3. Results and Discussion

**Figure 1:**
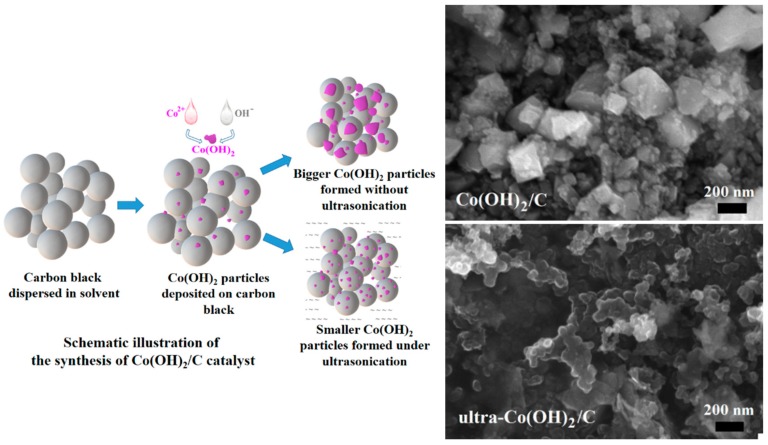
Fabrication of Co(OH)_2_/C, ultra-Co(OH)_2_/C and the scanning electron microscopy (SEM) images of the catalysts.

Figure 1 shows the preparation of the Co(OH)_2_/C catalysts without/with ultrasonication, and the morphologies of the resulting catalysts. Once the solutions of Co(NO_3_)_2_ and KOH were dropped into the suspension containing carbon black, the Co^2+^ ions immediately reacted with the OH^−^ ions to form the Co(OH)_2_ precipitates. These precipitates were deposited onto the surface of carbon black to yield the Co(OH)_2_/C catalyst.

During the initial stage of precipitation, the Co(OH)_2_ particles were small and had greater surface Gibbs free energies. The particles spontaneously assembled to form bigger crystals under quiet conditions. However, crystal growth was destroyed once ultrasonication was imposed on the reaction system. The SEM images in Figure 1 show that the hexagonal crystals of Co(OH)_2_ with size of approximately 400 nm can be observed in the Co(OH)_2_/C catalyst. The Co(OH)_2_ particles were bigger than that of carbon black, and they were not well incorporated with each other. However, only spherical particles with sizes of less than 50 nm could be observed, and no obvious Co(OH)_2_ particles with regular shapes were found in the ultra-Co(OH)_2_/C prepared with the assistance of ultrasonication. The results indicate that Co(OH)_2_ crystal could not grow well because of the disturbance caused by ultrasonication during precipitation. The Co(OH)_2_ precipitates existed as small particles, and they were absorbed on the surface of carbon black. Thus, better distribution and incorporation with carbon black substrate was found in ultra-Co(OH)_2_/C.

**Figure 2:**
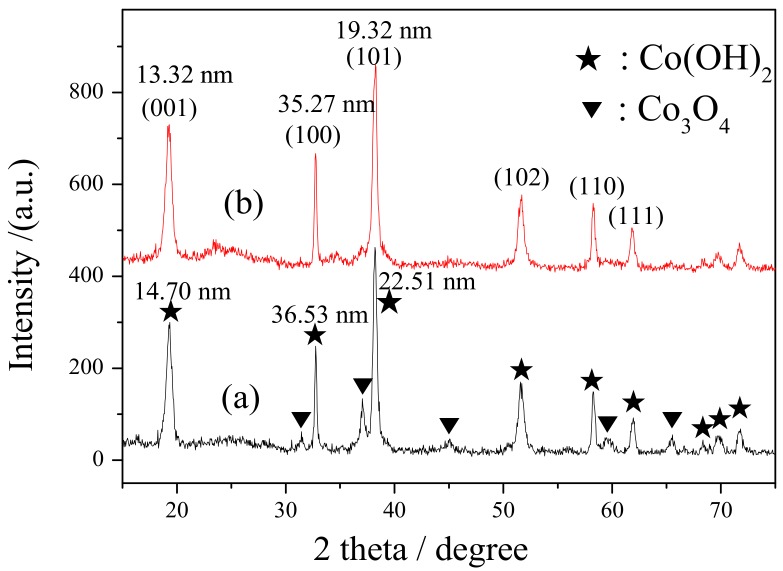
X-ray diffractometer (XRD) patterns of the (**a**) Co(OH)_2_/C and (**b**) ultra-Co(OH)_2_/C catalysts.

Figure 2 shows the XRD patterns of Co(OH)_2_/C and ultra-Co(OH)_2_/C. The cobalt element mainly existed as Co(OH)_2_ (JCPDS No. 30-0443) in the catalysts. The crystallite sizes of Co(OH)_2_ were calculated by the Scherrer Equation from three lattice planes (001), (100), and (101), and they were listed in the XRD patterns. Apparently, the crystallites were also refined by ultrasonication.

**Figure 3:**
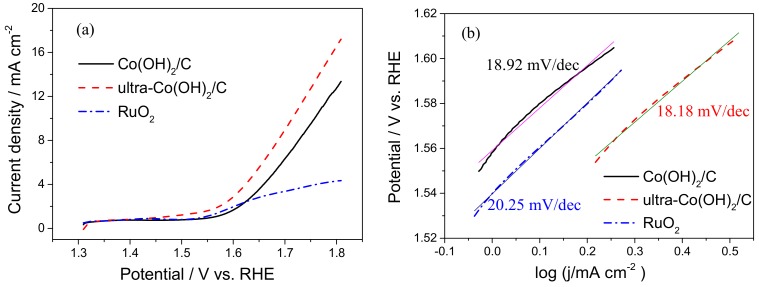
(**a**) Linear sweep voltammetry curves and (**b**) Tafel plots of ultra-Co(OH)_2_/C, Co(OH)_2_/C, and RuO_2_ catalysts in 0.1 M KOH.

The electrocatalytic performance of the catalysts to the OER was investigated in 0.1 M KOH solution. Figure 3a shows the linear sweep voltammetry (LSV) curves of the OER on the catalysts of Co(OH)_2_/C, ultra-Co(OH)_2_/C, and pure commercial RuO_2_. The OER onset potential of RuO_2_ was approximately 1.52 V, while those of Co(OH)_2_/C and ultra-Co(OH)_2_/C were approximately 1.54 V. The similar potentials show that the OER catalytic activity of Co(OH)_2_ was close to the OER activity of the benchmark RuO_2_ catalyst. However, the OER current densities of the three catalysts were different from each other. At the same potential of 1.66 V, the OER current density was 6.16 mA cm^−2^ on the ultra-Co(OH)_2_/C, which was greater than the 4.05 mA cm^−2^ of the Co(OH)_2_/C and the 2.95 mA cm^−2^ of RuO_2_. The improvement in catalytic performance of ultra-Co(OH)_2_/C can be attributed to its smaller particle size and better conductivity.

**Figure 4:**
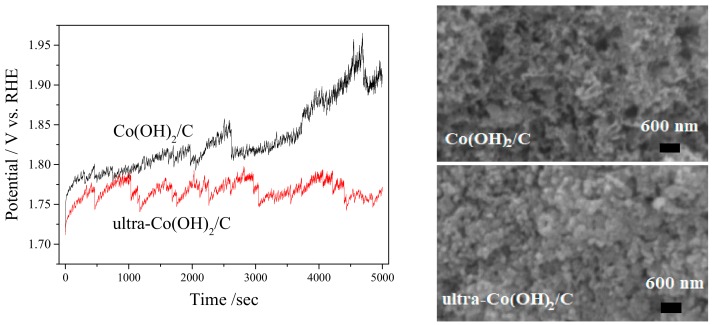
Chronopotentiometry curves of ultra-Co(OH)_2_/C and Co(OH)_2_/C catalysts at a current density of 10 mA cm^−2^ in 0.1 M KOH, and SEM images after catalyzing OER for 5 h.

The LSV curves were converted to Tafel plots and fitted by the Tafel equation (*η* = *a* + *b*lg*j*, where *η* is the overpotential, *j* is the current density, and *b* is the Tafel slope) to examine the catalytic kinetics of the catalysts, as shown in Figure 3b. As expected, the ultra-Co(OH)_2_/C showed a Tafel slope of 18.18 mV/dec, which was less than the 18.92 mV/dec of the Co(OH)_2_/C and 20.25 mV/dec of RuO_2_. Thus, the ultra-Co(OH)_2_/C could achieve higher current density at a lower overpotential than the Co(OH)_2_/C and RuO_2_ catalysts. Figure 4 presents the OER performed at 10 mA cm^−2^ of current density, and the SEM imagines of the two catalysts after catalyzing OER for 5 h. The electrode potential of ultra-Co(OH)_2_/C remained stable at a lower level in the OER process. However, the overpotential of Co(OH)_2_/C catalyst was greater, and it increased with the reaction time. As shown in the SEM images, the hexagonal crystals of Co(OH)_2_ in Co(OH)_2_/C catalyst disappeared after the OER. The broken crystals of Co(OH)_2_ in the OER process deteriorated the catalysis performance of the Co(OH)_2_/C catalyst.

These results indicate that the ultra-Co(OH)_2_/C catalyst has better OER catalytic performance than the Co(OH)_2_/C catalyst. This characteristic was due to the fact that the Co(OH)_2_ particles were smaller in the ultra-Co(OH)_2_/C catalyst than in the Co(OH)_2_/C catalyst without the disturbance from the ultrasonication during precipitation. Consequently, the Co(OH)_2_ in the ultra-Co(OH)_2_/C catalyst had a greater electrochemical specific surface area and more exposed active sites to catalyze the oxygen evolution reaction.

**Figure 5:**
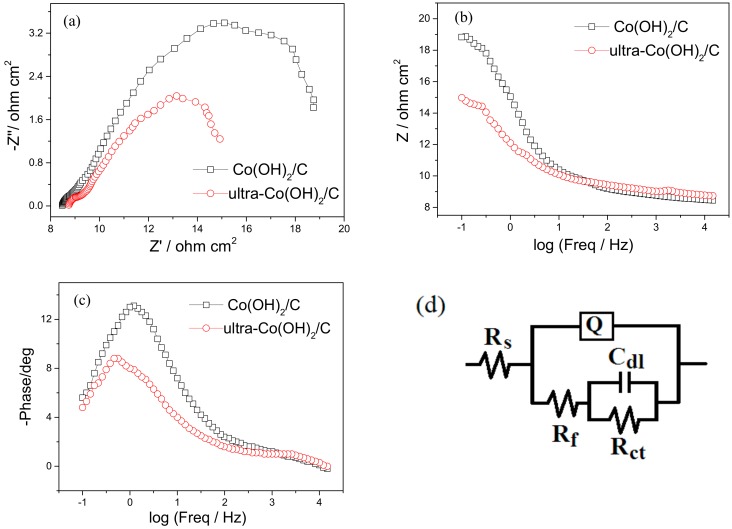
Electrochemical impedance spectra of Co(OH)_2_/C and ultra-Co(OH)_2_/C catalysts, (**a**) Nyquist curves; (**b**) Bode plots of Z versus frequency; (**c**) Bode plots of phase angle versus frequency; and (**d**) equivalent circuit model.

To further investigate the charge transfer kinetics during the OER, electrochemical impedance spectroscopy (EIS) was performed at 1.66 V of potential, at which OER were catalyzed both on the two catalysts. Figure 5a shows that the Nyquist curves of the Co(OH)_2_/C and ultra-Co(OH)_2_/C catalysts were composed of a small capacitive semi-circle at the high-frequency region, and a large capacitive semi-circle at the low-frequency region. The smaller impedance of the ultra-Co(OH)_2_/C catalyst (Figure 5b) corresponded to better conductivity. Two peaks existed in the Bode plots of the phase angle versus frequency, as shown in Figure 5c. Thus, the impedance spectra could be fitted by an equivalent circuit model with two time constants, as shown in Figure 5d. Herein, R_s_ represents the resistance of the electrolyte between the reference and working electrodes. R_f_ is the resistance of the catalyst itself. Q is the constant phase element in the catalyst. R_ct_ is the electron transfer resistance in the OER process. C_dl_ is a double-layer capacitance on the catalyst surface.

The values of the equivalent circuit elements were fitted using ZSimpDemo software (3.30d, Ann Arbor, MI, USA). Among of these elements, Co(OH)_2_/C and ultra-Co(OH)_2_/C have *R*_f_ values of 4.063 ohm cm^2^ and 3.205 ohm cm^2^, respectively, and corresponding *R*_ct_ values of 8.448 ohm cm^2^ and 5.487 ohm cm^2^. The reduced resistances reveal the smaller obstacle in the OER. Meanwhile, the 0.0173 F cm^−2^ of *C*_dl_ of ultra-Co(OH)_2_/C is greater than the 0.0073 F cm^−2^ of Co(OH)_2_/C. This result indicates the greater specific surface area and the greater number of active sites of ultra-Co(OH)_2_/C to facilitate the OER.

## 4. Conclusions

An OER catalyst ultra-Co(OH)_2_/C was fabricated by a parallel flow precipitate method with the assistance of ultrasonication. The growth of regular hexagonal crystals of Co(OH)_2_ was controlled using ultrasonication, and they decreased from approximately 400 nm to less than 50 nm. The smaller sizes of Co(OH)_2_ particles exhibit more active sites on the catalyst surface while they promote the formation of a more stable catalytically active film, upon mixing with carbon black. Due to these reasons, the ultra-Co(OH)_2_/C showed better OER catalytic activity with an onset potential of 1.54 V and a Tafel slope of 18.18 mV/dec, which ensured that the ultra-Co(OH)_2_/C worked well at a more stable and lower potential in the actual OER process. The conductivity of the catalyst was also improved because of the smaller particle of Co(OH)_2_ and the better incorporation with carbon black. The resistance *R*_f_ of the ultra-Co(OH)_2_/C itself and the electron transfer resistance *R*_ct_ during the OER were reduced by 21% and 35% compared to those observed in the case of Co(OH)_2_/C, without the assistance of ultrasonication, leading ultimately to improved catalytic performance in the oxygen evolution reaction.
